# Erie County Medical Society Dinner

**Published:** 1870-01

**Authors:** 


### Erie County Medical Society Dinner.

As a matter for future reference, and as a fact in history, it should be distinctly stated, that Erie County Medical Society does not, and probably cannot, eat dinner; it is never hungry, and will not eat if it is.

Resolutions for a dinner, and committees to provide dinner, and various suggestions about a Society dinner, are often made, and made all very well, but no real dinner will be needed.

